# Prevalence and Associated Factors of Diabetic Retinopathy in Type 2 Diabetes Mellitus Patients at a Specialized Hospital in Asmara, Eritrea: A Cross‐Sectional Study

**DOI:** 10.1155/jdr/5768546

**Published:** 2025-12-07

**Authors:** Oliver Okoth Achila, Millen Ghebretinsae, Abraham Kidane, Michael Simon, Shewit Makonen, Yohannes Rezene, Eyob Garoy, Oluwafemi Adekunmi Ibrahim, Yonas Tesfagabr Abraham, Robel Habte, Habtemichael Mulugeta Teklemariam, Samuel Tekle Mengistu

**Affiliations:** ^1^ Department of Clinical Laboratory Sciences, Asmara College of Health Sciences (ACHS), Asmara, Eritrea, uoa.edu.er; ^2^ Department of Ophthalmic Unit, Asmara College of Health Sciences (ACHS), Asmara, Eritrea, uoa.edu.er; ^3^ Department of Internal Medicine, Orotta School of Medicine and Dentistry, Asmara, Eritrea; ^4^ Department of Community Medicine, Orotta School of Medicine and Dentistry, Asmara, Eritrea; ^5^ Department of Internal Medicine, Tessenay Hospital, Ministry of Health Gash Barka Branch, Tesseney, Eritrea; ^6^ Nakfa Hospital, Ministry of Health Northern Red Sea branch, Nakfa, Eritrea

## Abstract

**Background:**

Diabetic retinopathy (DR) is a progressive microangiopathy affecting the retinal microvasculature and is a leading cause of preventable blindness in adults globally. Despite its significance, DR remains under‐investigated in sub‐Saharan Africa. This study was aimed at determining the prevalence of DR and its associated factors in Type 2 diabetes mellitus (T2DM) patients attending a specialized hospital in Asmara, Eritrea.

**Methods:**

A cross‐sectional study was conducted involving 309 T2DM patients from the diabetes follow‐up clinic at Halibet Referral Hospital, Asmara. Data were collected through structured questionnaires, clinical records, and biochemical analysis of blood samples. Ophthalmologic evaluation for DR was performed by eye specialists using ophthalmoscopy. Statistical analysis, including logistic regression, was performed to identify factors associated with DR.

**Results:**

The mean age of the participants was 57.8 ± 11.47 years, with an average disease duration of 12.14 ± 7.42 years. The prevalence of DR was 39.5% (95% CI: 34.03–44.9). Among these, 17.6% (95% CI: 11–25.1) had vision‐threatening DR. Factors significantly associated with DR included reduced estimated glomerular filtration rate (eGFR < 60 mL/min/1.73 m^2^) (aOR = 1.84, 95% CI: 1.05–3.20; *p* = 0.033), insulin use (aOR = 0.523, 95% CI: 0.295–0.93; *p* = 0.039), and longer duration of diabetes (> 8 years) (aOR = 0.24, 95% CI: 0.006–0.935; *p* = 0.040). Other factors associated with DR in the adjusted model were abnormal waist circumference (aOR = 0.298, 95% CI: 0.11–0.83; *p* = 0.021) and C‐reactive protein positivity (aOR = 2.65, 95% CI: 1.05–6.66; *p* = 0.039).

**Conclusions:**

The prevalence of DR among T2DM patients in Eritrea is high. Longer disease duration, reduced eGFR, and insulin use were significantly associated with DR. Early detection and management of these risk factors may help mitigate the burden of DR in diabetic patients.

## 1. Introduction

Diabetic retinopathy (DR) is a progressive microangiopathy involving the retinal microvasculature and is one of the leading causes of visual impairment (VI) or preventable blindness among working‐aged adults worldwide [1, 2]. In the recent past, a meta‐analysis involving 35 studies conducted worldwide estimated that the pooled prevalence of any DR is 35.4% [2]. A more granular analysis demonstrated that 17 million have proliferative diabetic retinopathy (PDR), 21 million had diabetic macular edema (DME), and 28 million had vision‐threatening diabetic retinopathy (VTDR) [2]. In addition, age‐standardized estimates of the prevalence suggest that DR‐related blindness and moderate and severe vision impairment (MSVI) were higher in low‐ and medium‐income countries (LMICs) in sub‐Saharan Africa (SSA) and South Asia [3]. On the whole, the VI associated with poorly managed diabetes mellitus (DM) may have multiple negative effects—reduced health‐related quality of life (HRQL) [4]. Aside from the personal impact, economic costs (e.g., direct expenditures and lost productivity) associated with VI are substantial [5].

At present, DR is not regarded as a leading cause of blindness in SSA—responsible for about 2.8% of blindness [5]. However, the incidence and prevalence of VTDR complications (including DR) are projected to increase—a correlate of the projected increase in T2DM cases in SSA [6].

Therefore, it can be asserted that the projected increase in DR will affect multiple countries in SSA with neither the ability nor the organization to deliver the necessary care [6]. Multiple publications also indicate that services for detection and management of DR in SSA are largely rudimentary and uncoordinated [4, 7]. Moreover, under‐diagnosis of both DM and DR, low awareness, inadequate referral systems, nonexistent systematic screening programs for eye pathologies, limited access to imaging technology and tests (e.g., spectral domain optical coherence tomography (SD‐OCT) and fundus fluorescein angiography (FFA), among others) and a dearth of eye care professionals (ophthalmologists, optometrists opticians, etc.) currently limit the ability of these countries to manage this rapidly evolving crisis [2, 4, 5, 7, 8].

In point of fact, DR is both a treatable and often preventable condition and the benefits of early detection are well established [4, 8]. Indeed, there is a strong consensus that regular screening for DR, timely treatment of patients with PDR (laser photocoagulation or vitrectomy), and more aggressive management of DM can reduce the proportion of patients presenting with VTDR [7]. Furthermore, data on the prevalence of DR (particularly VTDR) and associated risk factors may have practical utility in prioritizing, designing, and initiating intervention programs [9]. Existing literature also indicates that the presence of DR correlates with microcirculatory dysfunction in other organ systems [9–12]. To emphasize this point, the retina is the only point in the body where the condition of the systemic microcirculation can be accessed or imaged (noninvasively). Consequently, assessment of the frequency of DR in DM patients is widely considered a useful marker of cardiovascular disease (CVD) risk [12]. Finally, it can also be regarded as a surrogate measure of how well a healthcare system is performing with respect to the overall management of DM [7].

In Africa, data on the frequency of DR and/or associated risk factors is severely limited—a proposition that is true for Eritrea. Commenting on this issue, the recent Lancet and Endocrinology Commission report on DM in SSA decried the severe lack of reliable data on multiple aspects of T2DM, including complications/comorbidities in SSA [10]. Therefore, this study fills significant gaps in the knowledge base, specifically in the clinical epidemiology of DR in a cross‐section of T2DM patients in a specialised referral institution in one of the LMICs. The focus on specific clinical, anthropometric, and biochemical parameters is also unique and was designed to uncover patterns of known modifiable and unmodifiable risk factors that are predominant in this setting.

## 2. Participants and Methods

### 2.1. Study Design and Setting

This was a cross‐sectional hospital‐based study, undertaken at Halibet Regional Referral Hospital in Asmara, Eritrea, from February 2020 to June 2020. The selection of this hospital was informed by several considerations. Eritrea′s highly centralized health system provides specific services, such as chronic care, in select facilities. Halibet and Haz Haz are the only facilities in Asmara with DM clinics and follow‐up services. Therefore, Halibet has the largest pool of DM patients in Eritrea, receiving patients referred from other health facilities and self‐referred patients from across the country. This makes the hospital a key site for profiling the DM population in Eritrea. The patients are managed by a multidisciplinary team comprising general practitioners, pharmacists, and nurses during scheduled visits.

### 2.2. Sample Size Collection

#### 2.2.1. Data Collection

This study was based on secondary data extracted from patient records, as previously described [13], with additional information collected from patients and laboratory analyses. Variables obtained from patient charts included DM status, comorbidities (such as hypertension (HTN)), duration of DM, antidiabetic drug regimens, and CVD. A questionnaire incorporating queries on socioeconomic, anthropometric, lifestyle, and clinical factors was also employed to gather further details. Additional laboratory analysis of blood samples was conducted to supplement the data.

#### 2.2.2. Calculation of Sample Size, Recruitment, and Selection of Participants

The sample size was calculated as previously described [13]. Information from clinic records provided the sampling frame, and every second patient visiting the facility during the study period was randomly selected. Eligible participants included patients aged ≥30 years with Type 2 DM. Exclusion criteria involved Type 1 DM patients, hospitalized patients, those unwilling to provide consent, and individuals with specific psychiatric or mental health conditions. Presence or absence of retinopathy among the T2DM patients is the dependent variable, whereas the other variables are considered independent variables for this study.

The steps followed in primary data collection were as follows:

• Step 1: Use the Cochran formula to calculate the initial sample size.

As before, for a 95% confidence level, *p* = 0.5, and margin of error *e* = 0.05:

n₀=1.962·0.5·10.5−/0.052.n₀=3.84160.50.5··/0.00250.96040.0025384.16=/=.

• Step 2: Apply the finite population correction for *N* = 1032.

We now adjust the initial sample size for the finite population of 1032:

n=n₀/1+n₀−1/N.

Substitute *n*₀ = 384.16 and *N* = 1032:

n=384.16/1+384.161−/1032.n=384.16384.16384.161.3713280.13/1+383.161032/=/10.3713+=/≈.

• Step 3: Adjust for a 10% nonresponse rate.

Now, adjust the sample size by increasing it by 10% to account for possible nonresponse:

n_final=28028028308+2800.10×=+=.

#### 2.2.3. Measures and Operational Definitions

Outcome measures (dependent variables) and independent variables were defined as follows:

• **Lifestyle/behavioral factors:** Lifestyle factors such as alcohol consumption, dieting, and tobacco use (smoking) were defined according to previous studies ([13]).
• **Anthropometric measurements:** General obesity was assessed using body mass index (BMI), calculated as (weight in kg)/(height in m^2^). Waist circumference (WC), a marker of abdominal obesity (visceral adiposity (VAT)), was measured as per standard procedures [14]. Abnormal WC was defined as > 80 cm for women and > 94 cm for men, while the WHO criteria were used for BMI categorization: overweight (BMI ≥ 25.0 and ≤ 29.9 kg/m^2^) and obesity (BMI ≥ 30 kg/m^2^) [15]. DM duration was established by subtracting the age at diagnosis from the present age.

#### 2.2.4. Laboratory and Clinical Chemistry Measurements

Clinical chemistry tests were conducted at Sembel Hospital. After ≥ 8 h of fasting, 5 mL of blood was drawn from the median cubital vein, subdivided into appropriate biochemistry tubes, and analyzed within 3 h. The Latex test (Cortes Diagnostic, Inc.) was used to analyze C‐reactive protein (CRP). The Beckman Coulter AU480 Chemistry Analyzer was employed for the following: lipid panel (total cholesterol (TC), triacylglycerol (TG), and high‐density lipoprotein cholesterol (HDL‐C)), fasting plasma glucose (FPG), and hemoglobin A1c (HbA1c). Low‐density lipoprotein cholesterol (LDL‐C) was calculated using the Friedewald equation (LDL = non − HDL − TG/5, where non − HDL = TC − HDL − C). The categories for TC, TG, HDL‐C, LDL‐C, FPG, and HbA1c were based on American Diabetes Association (ADA) guidelines [16]. Abnormal TG/HDL was defined as values ≥ 3.0, and abnormal TC/HDL ratios as > 5.0 for men and > 4.4 for women.

#### 2.2.5. Estimated Glomerular Filtration Rate (eGFR)

The modification of diet in renal disease (MDRD) equation was used to calculate eGFR: eGFR = 186 × [serum creatinine (mg/dL)]^−1^. ^154^ × (age)^−0^. ^203^ × (0.742 if female). Reduced eGFR was defined as ≤ 60 mL/min/1.73 m^2^.

#### 2.2.6. Blood Pressure (BP)

BP was measured using a well‐calibrated digital sphygmomanometer (MDF Lenus Digital Blood Pressure Monitor), following WHO guidelines. Three BP measurements, each taken 5 min apart, were recorded, with the average of the second and third readings used as the participant′s BP. HTN was defined as a diastolic blood pressure (DBP) ≥ 90 mmHg or systolic blood pressure (SBP) ≥ 140 mmHg, a previous HTN diagnosis, or self‐reported antihypertensive medication use.

#### 2.2.7. Fundoscopic (Ophthalmoscopic) Examination

Dilated‐pupil fundus examination (DFE) was performed using a slit‐lamp ophthalmoscope (Zeiss SL 115 Classic, Carl Zeiss Meditec AG). Retinopathy was graded according to the International Clinical Diabetic Retinopathy Severity Scales [17], with the grade based on the worst eye or the only gradable eye [18].

#### 2.2.8. Data Analysis

Data were entered by a single individual and independently reviewed for accuracy by several investigators. Statistical analysis was performed using SPSS (Version 26.0; IBM, Chicago, IL). Descriptive statistics were calculated for continuous variables, with data presented as mean ± standard deviation (SD) or interquartile range (IQR). Depending on data distribution, means or medians were compared using one‐way analysis of variance (ANOVA) or the Kruskal–Wallis test, respectively. Tukey′s HSD test was used for post hoc analysis. The Chi‐square test or Fisher′s exact test evaluated associations between categorical variables. Group differences in continuous variables across DR severity categories (no DR, mild NPDR, moderate NPDR, and severe NPDR/PDR) were assessed using one‐way ANOVA or Kruskal–Wallis tests, while categorical variables were compared using Chi‐square or Fisher′s exact tests. As these analyses were intended to compare distributions across groups rather than to test for a linear trend, overall *p* values are reported instead of *p*‐for‐trend. Binary logistic regression was employed to identify factors associated with retinopathy, with a backward stepwise technique (backward: log‐likelihood ratio) used to determine the final multivariate model. Odds ratios (ORs) and 95% confidence intervals (CIs) were reported, with statistical significance set at *p* < 0.05.

#### 2.2.9. Ethical Approval and Consent to Participate

Ethical approval was obtained from the Eritrean Ministry of Health Research Ethical Committee. Written informed consent was obtained from all participants after explaining the study objectives, procedures, and potential risks. Participants were also informed of their right to withdraw from the study at any time and assured of the confidentiality and integrity of their data.

## 3. Result

Table 1 presents a description of the patient’s characteristics. A total of 309 T2DM patients were included in this analysis. The proportion of males to females was 163 (52.8%) versus 146 (47.2%). The mean (± SD) age of the study participants was 57.8 ± 11.47 (95% CI (56.52–59.1)) years and the median age ± IQR was 58 (51–66) years. The estimated average (± SD) duration of DM was 12.14 ± 7.42 years. Analysis of the data with respect to specific age bands demonstrates that 16 (5.2%) of the study participants were < 40 years; 170 (55.0%) were within 40–60 years age range. The rest (123 (39.8%) were in the > 60 years age category. Regarding specific parameters, 111 (35.9%) of the participants had a close family member with DM; 237 (76.7%) had HbA1c > 7*%* (mean: 8.72*%* ± 1.23*%*); 133 (43.0%) had HTN; 114 (36.9%) had eGFR (< 60 mL/min/1.73 m^2^). Additional information is presented in Table 1.

**Table 1:** Description of patient’s characteristics.

**Characteristics**	**Frequency (** **N** **)**	**Percentage (%)**
Age (years)		
< 40 years	16	5.2
40–60 years	170	55.0
> 60 years	123	39.8
Diabetes duration		
< 3 years	17	5.5
3–8 years	106	34.4
> 8 years	156	60.2
Sex (male, %)	164	53.1
Level of education		
No formal education	47	15.2
Primary	113	36.6
Secondary	98	31.7
Tertiary	51	16.5
Family member with DM	111	35.9
Dieting (no)	48	15.5
Waist circumference > 94 cm (men) & > 80 cm (women)	218	70.6
Body mass index (BMI) > 25 kg/m^2^	133	43.6
Smoking status (never, %)	294	95.1
Insulin injection (yes)	104	33.7
Fasting plasma glucose (FPG) >125 mg/dL	217	70.5
Hemoglobin A1c (HbA1c) > 7*%*	237	76.7
Hypertension (yes)	133	43.0
Systolic blood pressure (> 140 mmHg)	52	16.8
Diastolic blood pressure (> 90 mmHg)	13	4.2
Pulse pressure > 60	48	15.5
eGFR (< 60 mL/min/1.73 m^2^)	114	36.9
C‐Reactive protein (+ve)	35	11.3
Triglycerides (> 150 mg/dL)	174	56.3
Total cholesterol (> 200 mg/dL)	188	62.7
HDL cholesterol (50 mg/dL women and 40 mg/dL men)	137	44.3
LDL cholesterol (>130 mg/dL)	148	47.9
TC/HDL > 5.0 (men) & >4.4 (women)	292	5.2
TG/LDL > 3.0	140	45.3
Medications		
Insulin	122	39.5
Glibenclamide	142	46
Glimepride	38	12.3
Severe NPDR and PDR	21	7

*Note:* Values are expressed as number (%) or mean (standard deviation).

Abbreviations: DR, diabetic retinopathy; eGFR, estimated glomerular filtration rate; HbA1c, hemoglobin A1c; HDL‐C, high‐density lipoprotein cholesterol; LDL, low‐density lipoproteins; NPDR, nonproliferative diabetic retinopathy; TC, total cholesterol; TG, triacylglycerol.

### 3.1. Assessment of DR

Description of patient characteristics stratified by DR status is shown in Table 1. Among the cohort of 309 patients, one patient was blind and six patients failed to present themselves for DR evaluation. Overall, 122 (39.5%) presented with DR and 21 (17.6%) of the patients with DR had VTDR (severe NPDR and PDR). Separately, the data show that patients with any DR compared with those without DR had a longer duration of DM (*p* ≤ 0.001). Patients without DR also presented with a lower systolic blood pressure (SBP) 125.9 (±19) mmHg compared to those with any DR (mild NPDR; 133 (±21.1) mmHg; moderate NPDR 127 (±20) mmHg; and severe NPDR and PDR, 135 (±17) mmHg; *p* = 0.048). The mean value of eGFR also differed significantly between the disparate DR and no DR status groups. See Table 2 for additional information.

**Table 2:** Clinical characteristics of patients with diabetes stratified by retinopathy status.

**Variable**	**No DR**	**Mild NPDR**	**Moderate NPDR**	**Severe NPDR and PDR**	**p** **trend**
*N* (%)	182 (60.7)	77 (24.99)	21 (7.0)	21 (7.0)	
Age, mean ± SD	57 ± 12.3	59.5 ± 9.8	58.1 ± 7.7	58.3 ± 11.8	0.577^a^
Gender					
Male	93 (51.1)	45 (58.4)	12 (57.1)	10 (50)	0.71 (1.4)
Feamle	89 (48.9)	32 (41.6)	9 (42.9)	10 (50)	
Educational level					
No formal education	24 (13.2)	12 (15.6)	6 (28.6)	3 (15)	0.45 (8.8)
Primary	70 (38.5)	24 (31.2)	6 (28.6)	7 (35)	
Secondary	62 (34.1)	23 (29.9)	7 (33.3)	5 (25)	
Tertiary	26 (14.3)	18 (23.4)	2 (9.5)	5 (25)	
Marital status					
Single	23 (12.8)	6 (7.8)	2 (9.5)	1 (5.3)	0.5 (2.1)
Married	156 (87.2)	71 (92.2)	19 (90.5)	18 (94.7)	
Employment					
Employed	94 (51.6)	38 (49.4)	13 (61.9)	14 (70)	0.32 (3.5)
Not employed	88 (48.4)	39 (50.6)	8 (38.1)	6 (30)	
Duration of diabetes (years)	10.67 ± 7.01	14.63 ± 7.8	14.3 ± 7.2	14 ± 1.66	0.001
Family history of DM					
Yes	70 (38.5)	25 (32.5)	8 (38.1)	5 (25)	0.58 (1.9)
No	112 (61.5)	52 (67.5)	13 (61.9)	15 (75)	
Hypertension status					
Hypertensive on treatment	65 (35.7)	29 (37.7)	11 (52.4)	6 (30)	0.26 (7.6)
Hypertensive not on treatment	2 (1.1)	4 (5.2)	0	1 (5)	
Not hypertensive	115 (63.2)	44 (57.1)	10 (47.6)	13 (65)	
SBP (mmHg)	125.9 ± 19	133 ± 21.1	127 ± 20	135 ± 17	0.048
DBP (mmHg)	80 ± 8.3	80.1 ± 6.4	82 ± 6.2	81.1 ± 7.6	0.218
Pulse pressure	47.0 ± 16.51	52.9 ± 19.24	45.5 ± 18.8)	54.0 ± 16.8)	0.080
BMI (kg m^-2^)	24.6 ± 3.8	25.3 ± 4.2	22.7 ± 5.9	25.9 ± 6.8	0.100
Waist circumference					
Male	94.15 ± 8.84	94.41 ± 9.41	93.75 ± 5.86	96.10 ± 11.44	0.923
Female	93.37 ± 12.14	99.75 ± 10.88	92.22 ± 7.53	95.30 ± 9.57	0.055
HbA1c	8.65 ± 1.22	8.9 ± 1.11	8.82 ± 1.06	8.7 ± 1.43	0.542
Fasting plasma glucose (mg/L)	172 ± 73.9	183.6 ± 82	164 ± 60	161 ± 62	0.643
eGFR (mL min^-1^ 1.73 m^-2^)	87.7 ± 46.41	71.4 ± 33	77.4 ± 35.1	69.5 ± 30.5	0.003
HDL					
Men	43.95 ± 9.64	44.49 ± 9.57	41.17 ± 4.04	40.5 ± 9.68	0.568
Women	52.6 ± 12.23	53.0 ± 10.42	54.0 ± 10.12	48.89 ± 4.7	0.772
LDL	134 ± 42	135 ± 43	141 ± 31	123 ± 30	0.722
TG	206.1 ± 100	206.8 ± 138	174.7 ± 53	155.3 ± 79.4	0.550
TC	217.5 ± 50.0	222.9 ± 55	223 ± 36.1	198 ± 40	0.385
TG/HDL	4.8 ± 4.7	4.7 ± 2.6	3.9 ± 1.4	3.6 ± 2.06	0.736
TC/HDL	4.7 ± 1.08	4.74 ± 1.08	4.86 ± 0.64	4.57 ± 0.99	0.860
Medication/s					
Insulin	63 (35.6)	34 (45.3)	9 (42.9)	10 (3.4)	0.6
Glimpride	24 (13.6)	10 (13.3)	1 (4.8)	2 (10)	
Glibenclamide	90 (50.8)	31 (41.3)	11 (52.4)	8 (40)	
Insulin (IU/kg, mean ± SD)	0.47 ± 0.12	0.5 ± 0.11	0.52 ± 0.11	0.42 ± 0.13	0.2

*Note:* Data are means ± SD or median (interquartile range).

Abbreviations: BMI, body mass index; DBP, diastolic blood pressure; DR, diabetic retinopathy; eGFR, estimated glomerular filtration rate; HbA1c, hemoglobin A1c; LDL, low‐density lipoproteins; NPDR, nonproliferative diabetic retinopathy; SBP, systolic blood pressure; TC, total cholesterol; TG, triacylglycerol.

^a^An independent sample *t*‐test.

### 3.2. Relationship Between DR Status and Duration of Type 2 DM

**Figure 1:**
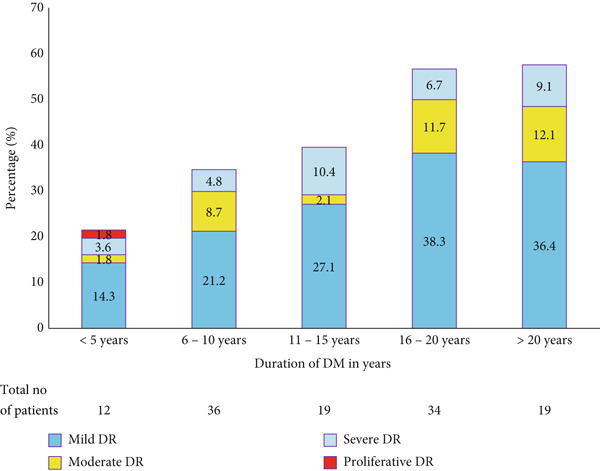
Classes of retinopathy by duration of Type 2 retinopathy.

The relationship between DR status and duration of DM is shown in Figure 1. In this analysis, the prevalence of DR increased with longer durations of DR. At the same time, a significant proportion of the patients with DR were diagnosed less than 5 years ago.

#### 3.2.1. Factors Associated With DR

A number of variables were associated with DR in the univariate and the adjusted multivariate model: no insulin injection (aOR = 0.523, 95% CI, 0.295–0.93; *p* < 0.039); eGFR (< 60 mL/min/1.73 m^2^) (aOR = 1.84, 95% CI, 1.05–3.20; *p* < 0.033); and duration of DM (> 8 years; reference) (<3 years; aOR = 0.24, 95% CI, 0.006–0.935, *p* < 0.040) (3–8 years; aOR = 0.441, 95% CI, 0.25–0.79, *p* < 0.006).

The factors which were only associated with DR in the adjusted model included: WC (reference: normal) (aOR = 0.298, 95% CI, 0.11–0.83; *p* < 0.021) and CRP (reference: positive) (aOR = 2.65, 95% CI, 1.05–6.66; *p* < 0.039). Reducing HDL‐C and increasing TC exhibited a tendency towards significance in this model (see Table 3).

**Table 3:** Univariate and multiple logistic regressions for risk factors of diabetic retinopathy in individuals with diabetes aged 0 years and older attending Halibet National Referral Hospital, Asmara, Eritrea (*N* = 309).

**Variables**	**Diabetic retinopathy (DR)**		**Crude model**		**Adjusted model**	
	**No DR,** **N** **(%)**	**DR,** **N** **(%)**	**COR (95% CI)**	**p** **value**	**aOR (95% CI)**	**p** **value**
Waist circumference (WC)						
Abnormal	146 (57.1)	112 (42.9)	1 (reference)	0.051	1 (reference)	**0.021**
Normal	18 (73.1)	7 (26.9)	0.332 (0.11–1.00)		0.298 (0.11–0.83)	
Insulin injection						
Yes	41 (47.1)	46 (52.9)	1 (reference)	0.066	1 (reference)	**0.026**
No	127 (63.5)	73 (36.5)	0.57 (0.31–1.04)		0.523(0.295–0.93)	
C‐reactive protein (CRP)						
Negative	146 (56.8)	111 (43.2)	1 (reference)	**0.014**	1 (reference)	**0.039**
Positive	22 (73.3)	8 (26.7)	3.52 (1.3–9.571)		2.65 (1.05–6.66)	
Age			0.99 (0.96–1.03)	0.734	—	—
Systolic BP (mmHg)	**—**	**—**	1.01 (1.00–1.031)	0.116	—	**—**
Diastolic BP (mmHg)	**—**	**—**	0.998 (0.96–1.04)	0.943	—	—
Sex					**—**	
Male	89 (56.7)	68 (43.3)	1 (reference)	0.45		
Female	79 (60.8)	51 (39.2)	1.31 (0.65–2.66)			
eGFR (MDRD) (mL/min/1.73 m^2^)						
> 60	71 (65.1)	38 (34.9)	1 (reference)	**0.026**	1 (reference)	**0.033**
< 60	97 (54.5)	81 (45.5)	2.14 (1.09–4.19)		1.84 (1.05–3.20)	
Hemoglobin A1c (HbA1c)					—	—
> 7%	124 (56.4)	96 (43.6)	1 (reference)	0.115	—	—
< 7%	44 (65.7)	23 (34.3)	0.58 (0.29–1.14)	—	—	—
BMI (Kg/m^2^)			1.01(0.94–1.08)	0.810	—	—
> 25	94 (59.5)	64 (40.5)	—	—	—	—
< 25	73 (58.4)	52 (41.6)	—	—	—	—
Duration of DM			—	—	—	—
< 3 years	13 (76.5)	4 (23.5)	0.25 (0.06–1.06)	0.059	0.24 (0.06–0.935)	**0.040**
3–8 years	70 (72.2)	27 (27.8)	0.48 (0.242–0.82)	**0.010**	0.441 (0.25–0.79)	**0.006**
> 8 years	85 (49.1)	88 (50.9)	1 (reference)	**0.013**	1 (reference)	**0.005**
HDL (mg/dL)	—	—	0.78 (0.315–1.91)	0.581	0.97 (0.941–1.00)	0.074
LDL‐C (mg/dL)	—	—	0.80 (0.325–1.97)	0.630	—	—
TC (mg/dL)	—	—	1.25 (0.51–3.09)	0.622	1.01 (999–1.01)	0.068
TG (mg/dL)	—	—	0.951 (0.79–1.14)	0.586	0.994 (0.99–0.999)	0.099

*Note:* Predicted probability is of membership to the presence of diabetic retinopathy. Symbols indicate significant association (*p* value < 0.05). The *p* values that are less than or equal to 0.05 are in bold.

Abbreviations: aOR, adjusted odds ratio; BMI, body mass index; COR, crude odds ratio; DM, diabetes mellitus; eGFR, estimated glomerular filtration rate; HDL, high‐density lipoprotein; LDL‐C, low‐density lipoprotein; MDRD, modification of diet in renal disease; TC, total cholesterol; TG, triacylglycerol.

## 4. Discussion

In general, there is a consensus that in early DR, a multifactorial treatment approach including laser photocoagulation can effectively be used to prevent loss of vision [18, 19]. However, the absence of visual loss or other overt symptoms in the early stages of DR minimizes the possibility of timely diagnosis. Therefore, regular screening is generally prescribed. In Eritrea, periodical funduscopic (retinal) examination of DM patients is not scheduled as needed. Moreover, evidence suggests that diagnostic or screening procedures are performed without proper systematization. Evidence, usually epidemiologic, has also demonstrated that lack of data on the clinical epidemiology of DR undermines intervention efforts in SSA. However, research has also demonstrated that there is considerable, context‐specific variation in the nature, pattern, and strength of known risk drivers (e.g., duration of DM, chronic hyperglycemia, and HTN) [8, 9]. In this paper, we report on the prevalence of DR in patients attending Halibet Referral Hospital (the largest DM follow‐up clinic in Asmara, Eritrea) and associated factors. This is in contrast to a previous study which profiled DM patients presenting with visual complications at a referral eye hospital in the country and was therefore subject to referral bias [19].

In all, the prevalence of DR was 39.5% (95% CI (‐)). Nearly all the patients had no previous eye examination. The study appears to corroborate the proposition that the frequency of DR in DM patients in SSA is disproportionately higher relative to that of patients from more affluent regions. Indeed, a high frequency of DR has been reported in several clinic‐based studies in specific settings in SSA: Cameroon, 40.3% [20]; Ethiopia 51.3% [21]; Malawi, 50.1% (95% CI 44.9–55.3) [18], Tanzania, 27.9% (95% [CI], 26.4%–29.5%) [22]; and Zambia, 55% [23]. In aggregate, a previous systematic review noted that DR affects 30.2%–31.6% of patients in SSA [2]. In contrast, data from western countries have reported relatively low rates, for example, USA, 28.5% (95% CI, 24.9%–32.5%) [24]. Further, 17.6% of the patients with DR had VTDR. The magnitude of VTDR reported in this study is relatively lower compared to values reported in a study from Zambia where 36% of study participants had VTDR [23]. Altogether, we have to highlight the conclusion from a past review of hospital‐based surveys from Eastern Africa (EA) which warned of the increasing prevalence of DR [7].

Interestingly, clinical epidemiological studies support the idea that DR data from SSA are non‐identical across replicate studies generating somewhat dissimilar, albeit overlapping, results [18]. A number of factors have been invoked to explain this phenomenon including variations in data quality, disparate study design procedures, data reporting formats, retinal examination techniques and grading systems [7]. The observed variability has also been explained with reference to differences in the impacts of specific drivers such as BP, HbA1c, gene–environment interactions, levels of serum lipids and sociocultural background (diet and lifestyle including physical activity), among others [20]. Much remains to be uncovered on the relationship between these variables and DR in the Eritrean population. Notwithstanding, the high frequency of DR in SSA may be a reflection of the fact that care for DM patients tends to be suboptimal [4]. This phenomenon has also been linked to multiple institutional and patient‐related shortcomings [4].

In general terms, researchers have argued that clinical epidemiologic studies can help in establishing consistent patterns of concordance of risk and protective factors. This proposition is particularly relevant in SSA. Interestingly, previous reports have noted that the clinical characteristics of patients in SSA, particularly patterns of risk factors associated with DR differ from Caucasian populations [7, 23]. This notwithstanding and in keeping with previous research [1, 21, 25]; we found a clear relationship between elevated SBP, insulin injection, eGFR, and duration since DM diagnosis and DR in this setting. These associations were established in both univariate and multivariable analyses. In other words, the predictive power of these variables was not affected by the other covariables. Importantly, the patterns of risk factors reported in this study are similar to those reported from a large cross‐sectional study designed to uncover the frequency of DR and VI in Zambia’s Copper belt province [23]. However, whether these findings reflect similarities (social, and level of access to healthcare, among others) is hard to uncover.

Apart from uncovering a relatively high frequency of nephropathy, our analysis also demonstrated that eGFR was significantly lower in patients with DR. This aligns with previous reports that have examined the relationship between DR and nephropathy [26, 27]. Importantly, surveys have reported that the co‐existence of DR and chronic kidney disease (CKD) is associated with increased all‐cause mortality [27]. The implications of these findings for LMIC like Eritrea are manifold. At the very least, the evidence suggests that patients with DR should also be referred for renal status evaluation. Unfortunately, most DM patients in Eritrea are rarely referred for such evaluation. What is more, the existing nonprogrammatic evaluations of renal function are limited to plasma blood urea nitrogen (BUN), Cre, and dipstick analysis of urine. Essential metrics such as eGFR estimates, quantitative albumin‐specific immunoassays, measurements of albumin/creatinine ratio (UACR), or other more advanced techniques of renal status evaluation are unavailable. For these reasons, we did not incorporate the presence of albuminuria or UACR in this study. In all, we endorse the proposition that the duration of DM, SBP, eGFR and insulin injection are important factors to consider in the implementation of successful DR screening and awareness‐raising programmes in this setting [22].

Additional factors which were associated with DR in the multivariable model included increasing age, increasing WC and CRP positivity. The association between these factors and DR has been documented in literature, albeit with varying degrees of strength [28–30]. In some studies, however, elevated concentrations of CRP are associated with specific abnormalities characterizing the metabolic syndrome (Mets), including obesity, low HDL‐C, high TG, and insulin resistance [31–33]. CRP is also an established prognostic marker in acute coronary syndrome (ACS) and can also be a risk marker of perivascular disease (PVD). Other reports have noted that high levels of CRP may also be associated with worse glycemic control [34]—an important risk mediator of DR. Surprisingly, a negative association between CRP positivity and DR was uncovered in this study. Of note is the fact that this association was only uncovered in the adjusted model thereby implicating the involvement of specific cofounders. Revisiting the issue on the connection between DR and CRP, a number of reviews/studies have concluded that the results are inconsistent with both positive and negative associations [8, 9, 31, 35].

In the current study, aberrant lipid profiles, HbA1c, BMI, sex, and smoking were not associated with DR. This finding is consistent with a number of studies which have noted that neither being overweight nor obesity confers an increased risk of DR [23, 36]. In contrast, some studies have reported a decreased incidence of DR in individuals with elevated BMI [37]. Interestingly, we found a link between WC and DR. This association has been reported in previous studies [38]. Further, it is important to note that multiple studies in diverse populations, unequivocally support an association between specific lipid parameters and DR. For instance, landmark longitudinal studies such as DCCT‐EDIC indicated that the severity of DR is negatively associated with HDL‐C and positively associated with TG [39]. Likewise, a recent large‐scale study failed to establish any relationship between DR and derangements in specific lipids [40]. As noted by prior reviews, the evidence for dyslipidaemia as a risk factor for DR is unconvincing and no single lipid measure has consistently been found to be associated with DR [9]. Irrespective, the high proportion of patients with lipid abnormalities should raise concern. Unfortunately, improved metabolic control of DM is frequently underemphasized by the ophthalmologist or other eye care specialists because changes in patient management are made by internists. This is the case in Eritrea. To remedy this situation, multifactorial intervention aimed at optimizing glycemic, lipid, and BP control has been proposed [30].

## 5. Limitations

This study is one of very few studies estimating the prevalence of DR and associated factors in SSA. However, results should also be considered in light of several additional limitations. For example, the study suffers from the limitations associated with cross‐sectional studies, including the inability to establish the temporal relationship between cause and effect. Unverifiable responses by respondents and other uncertainties may also be limiting, for example, information on alcohol consumption, dieting, and smoking status, among others. Importantly, information from this study should be used cautiously when making overly broad generalizations. For such generalization, larger, more rigorously designed studies are required. Last, but certainly not least, it is our opinion that this study provides crucial information on the burden of DR and associated factors in patients attending one of the major institutions responsible for DM management in Asmara, Eritrea.

## 6. Conclusion

In this study, the prevalence of DR among patients attending this facility was found to be high at 39.5%, with 17.6% of these cases classified as VTDR. This highlights a significant burden of DR within the studied population in Eritrea. Key factors associated with DR included longer duration since diagnosis, higher systolic blood pressure, reduced eGFR (eGFR < 60 mL/min/1.73 m^2^), and insulin use. Systemic metabolic abnormalities, such as increased WC, were also identified as contributing factors.

## 7. Recommendations

Considering these findings, we recommend that systematic screening for DR be institutionalized, especially targeting patients with the identified risk factors. Further research should aim to explore the variations in DR prevalence across different regions of Eritrea through both cross‐sectional and longitudinal studies, incorporating a broader spectrum of clinical and biochemical markers.

## Ethics Statement

Ethical approval was obtained from the Ministry of Health (MOH) Research Ethics and Protocol Review Committee (letter of reference: 02/05/20). All study procedures followed the recommendations of the Declaration of Helsinki Convention. In addition, consent to participate in the study was obtained from enrollees. This was obtained after provision of information on the objectives of the study, adverse effects, study protocols, and invasiveness of procedures. The participants were also counselled on the right to terminate their participation at any time. Information on confidentiality and data integrity was also provided.

## Consent

The authors have nothing to report.

## Disclosure

All authors gave final approval of the version to be published, have agreed on the journal to which the article has been submitted, and agree to be accountable for all aspects of the work.

## Conflicts of Interest

The authors declare no conflicts of interest.

## Author Contributions

All authors made a significant contribution to the work reported, whether that is in the conception, study design, execution, acquisition of data, analysis and interpretation, or in all these areas and took part in drafting, revising or critically reviewing the article.

## Funding

No funding was received for this manuscript.

## Data Availability

The data that support the findings of this study are available from the corresponding author upon reasonable request.
